# Miscellaneous

**Published:** 1858-06

**Authors:** 


### Miscellaneous.

### "CHEOPLASTIC" METAL—ITS PRETENSIONS AND MERITS.

BY B. W. WOOD, M. D., DENTIST,

*Late Associate Editor Southern Journal of Medical and Physical Sciences.*

The metallic alloy recently patented by one of the ex-professors of the Baltimore College of Dental Surgery, and strongly recommended to the profession by some of its most prominent members, especially by professors of that widely noted Institution, has naturally awakened much attention. Not conceiving it to be what is claimed for it, and observing that our dental journals have been remarkably silent on this side of the question, thereby, if not directly corroborating, seeming to acquiesce in the claims set forth, (although probably only waiting for further developments in the premises,) I have thought a somewhat extended examination into the subject might not be without interest. Upon its first announcment, backed by such respectable names, who could hardly be deceived, I confess to entertaining the highest hopes in regard to it; but failing to learn anything of its constituents, or to obtain a specimen by which to test its virtues for myself, either from the patentees or their agents, I began to feel some doubts, and concluded to wait for further light. I did not question, however, but that the metal which aspired to rivalry with gold and platina, would prove a valuable substitute for the inferior materials in use. But this hope has given way before a knowledge of its components, and the ordeal of experimental tests. Let us, by these lights, examine its actual merits—but let us first review some of the extraordinary claims which have been set up for it:

Prof. Blandy, in his circular to the profession, states at the outset, in general terms, as follows:

"This new process, of which I am the inventor, has become, through its adoption by many of the leading members, of the professiona fixed and reliable practice, in most cases to the exclusion of all other methods, and must be in its perfection universally preferred by both operator and patients." * * * "In no case nor under any circumstances, has the skillful application of Cheoplasty been attended with other than complete success."

After some further remarks he conveys the idea that by the discovery of some peculiar laws of "chemical *combination*" till now shrouded in obscurity, he has succeeded in transforming certain metals hitherto oxidable, subject to galvanic influences, &c., into a new metal with altogether different characteristics, and possessed indeed of every quality requisite for dental purposes. "That this cheoplastic metal" (he argues) "is capable of resisting oxidation under ordinary circumstances, and that it is free from galvanic influences, free from metallic taste, is not more strange than that certain combinations of metals produce different qualities than are found in their constituents, such as ductility, maleability, hardness" and the like.

What is thus asserted or implied is next backed by an imposing array of analogies, drawn from chemistry and metallurgy, well calculated to over-awe the skepticism of some and inspire the confidence of others, especially after the emphatic assurance—" I present facts alone, substantiated by the very highest and most *unquestionable* authorities."

It is to be remarked that although he speaks in this connection of tin—the chief constituent of this Cheoplastic metal, (although he does not reveal the fact,) of the strange "conduct of tin under the action of acids," &c., he does not venture to assert that tin alone is "capable of resisting oxidation under ordinary circumstances, free from galvanic influences," free from metallic taste" and the like; but leaves it to the full force of such objection, until, indeed, transmuted by his process of combination, into "Cheoplasty," it becomes at once exempt from oxidation, &c. He prepares us for the reception of such anomaly, by stating as a fact in chemistry, that, "metals easily acted upon alone, or in certain combinations, by one acid, in other conditions of alloy remain untouched." As an instance, he specifies the metals platina and bismuth, wherein he informs us that the action of hydrochloric acid, which he says is the "especial solvent of bismuth—upon this metal "is instantly arrested by the contact of a small piece of platina," and that the alloy of these metals "remains unaffected in this acid." But hydrochloric acid, instead of being the especial solvent of bismuth, has scarcely any action upon it; even when heated, its action is very slow and feeble, and it is none the less so when in connection with platina. Perhaps, however, he meant *nitric* acid, (which dissolves bismuth vehemently,) since it is stated in Piggot's *Dental Chemistry* and *Metalurgy* that the process of solution in this acid "is immediately arrested by laying a piece of platinum on the metal bismuth." But the truth is, that the action of nitric acid upon bismuth is not diminished but perceptibly increased by the contact of platina as any one may test for himself.

We might examine the validity of these paraded analogies throughout, were our object to point out the signal inaccuracies and perversions of chemical facts which they display, but we only intended to show the specious claims set up for this "*new* metal," by way of contrast with its naked merits.

The Circular proceeds—implying a great deal more than it directly avers. It speaks of the development of unlooked for properities in alloys from "a certain combination" of their components; of galvanic and electrical agencies on metals, of their precipitation from solutions, "Impure crystals" by the contact of other metals, "producing *pure crystalline* deposits," &c., all aiming to show the capability of inducing by apparently slight causes, as "mere mechanical contact of metals," a "wonderful inversion" of their properties, and that "under certain combinations, acids lose their power of attacking metals." It further informs us that "it is not uncommon in making assays, after abstracting one metal to add others in order to reach the desired purity," ("*purity*," mark the word,) and how the laws of affinity are consulted "so as to combine the added metal with the one retaining the metal *to be purified*," and how this is all to be done "at a precise temperature or failure results," and what neeessity there is for the greatest accuracy in the use of all re-agents, &c.

We are then told that the celebrated Dr. Brandt, long years, ago, stated that he thought time would prove, "that in the combination of metals, new metals would be produced of distinct qualities, of as peculiar characters as is found in a simple elementary metal;" "in other words," (continues the circular,) "from alloying to form a *chemical union*, so as its constituents are lost in the development of the one, not professing any relation to them—in fact a *new* metal, whose chemical and mechanical characters are different from all others."

Thus prepared for the belief that "this Cheoplastic may" indeed be the realization of all this, we have next the emphatic announcement that "*the metal is as pure to wear as the gold commonly used for dental purposes*," all from alloying tin with bismuth, antimony and a little silver, which prove to be its components. Then comes the customary "CAUTION" to the profession, "*against the impositions that have and will be practiced upon them*, by men who know nothing about metals or their chemical qualities;" that "*there is great danger in using impure metals in the mouth*," &c., &c.

To the circular is appended an array of recommendations more than corroborative of all the patentee himself ventures to claim for his metal. And, what is the most astonishing feature in the affair, these testimonials are mainly from men who have held the highest rank in our profession, as authors, as teachers, as professional guides, who have hitherto led the rest whithersoever they listed.

But first take the evidence of a professed chemist, Dr. Gideon B. Smith, of Baltimore, who affirms: "chemically considered the metal is perfect. No acids that can ever come in contact with it in the mouth, and none of the secretions of the mouth can have any effect upon it." But it does not appear that Dr. Smith had submitted it to any direct chemical test, except applying to it a current of sulphuretted hydrogen, and leaving it exposed to the common atmosphere of his laboratory, whose gases he says would have blackened pure silver; thus applying the test for *silver* to a compound whose chief component is *tin*, and whose quantum of silver is so small as to be inappreciable by such test. Why did he not submit it to re-agents capable of affecting tin, and then ascertain if any such re-agents were ever present in the mouth?

Next comes Prof. P. H. Austen, of the Baltimore Dental College, whom we have all admired for the utility, beauty, perspicuity, and general excellence of his writings. He implies much more than he clearly asserts, refers to his "favorable opinion" previously published, and says: "my experience since writing these communications, confirms me in the views there expressed." But he abstains from speaking directly of the chemical qualities of the metal.

Dr. Chas. W. Ballard of New York City, who, as a writer of spirit, independence and manly candor, has earned honorable fame in the profession, proclaims that this metal has, among other enumerated advantages, that of "*perfect purity* and *cleanliness*, equal in this respect to Allen's continuous gum-work, and superior to every other style of mounting artificial teeth." Did he mean in its property of resisting chemical contaminations, as probably every one would understand, or did he mean merely in the exclusion of substances from lodgment in interstices between plate and teeth? And if the latter, why did he not also instance ordinary tin-work as an exception ?

Dr. Wm. H. Dwinelle, prominent as a professor in the New York Dental College, who has written extensively on dental subjects, and won the reputation of an able and zealous advocate of almost every new method or process that has been submitted to the profession backed by respectability, adds the weight of his testimony to this: "After fully testing it," says he, " I unhesitatingly accord to it the following advantages"—among which he enumerates, " its non-corrosiveness, being equal to gold or platina in this respect. But he does not inform us of the means he employed in thus "*fully testing*" its virtues.

Dr. Edward Maynard, of Washingtom City, professor in the Baltimore Dental College, expresses himself "convinced" that the " invention will prove of immense benefit," but is frugal of words.

To close the array of testimony, (of which the foregoing forms a part,) we have lastly the high authority of Prof. C. A. Harris, (of the Baltimore Dental College,) who has long been held the oracle of the profession. He says he has adopted this method in his practice, and states as his conviction, from observation and experience, that "it possesses in a large majority of cases, decided advantages over every other heretofore practiced." This, it will be recollected, comes from one who has ever been foremost and most emphatic in deprecating the employment of base metals for dental purposes, or of any material whatever, capable of undergoing oxidation or corrosion in the mouth—particularly amalgam and *tin*. What better testimony could be had to the "purity" and " non-corrosiveness " of this alloy, which he here so strongly recommends, by example and precept, to the use of the profession?

Now, from such recommendation, and accumulation of evidence, might we not feel assured that Prof. Blandy, by some peculiar chemical manipulation with tin, had really produced a new metal, of very different chemical properties, fitted to stand side by side, or a little in advance of gold and platina; that indeed pure tin, which has been employed for a number of years in the casting of plates, and upon substantially the same process as Dr. Blandy's, but to be found wanting in the "great majority of cases," on account of its susceptibility to corrosion and oxidation in the mouth, and which the best authorities in the profession have concurred to condemn as a material for plugging, on the same grounds that this tin, in its purest form, proving itself to be impure, had now by Dr. Blandy's method of "purifying," by addition of other metals and "alloying to form a *chemical union*," been in fact transformed into a metal, "*as pure to wear*" (to say the least of it) "*as the gold commonly used for dental purposes?*"

But, what is the truth? What are the chemical properties of this metal, as revealed by experiment, and what its advantages or fitness for dental purposes?

In order to satisfy others and assure myself beyond the possibility of mistake, I subjected the metal upon a recent occasion, to the action of several re-agents most conveniently at hand, and although I can not say with Dr. Dwinelle, that I have "*fully* tested it," yet I think it will be sufficient to indicate its impurity in the mouth, its liability to corrosion, and inadequacy for dental use in the great majority of cases. The metal was Prof. Blandy's "genuine," bearing his patent-stamp, &c.

I will state in order the different re-agents employed, and their results, as noted down at the time; also, upon tin and a few other metals, where brought in comparison, since neither our dental works nor chemical text books are specific on this subject. In the first three cases the acids were purposely much diluted; the reactions corresponded with what is known of tin:

1. Dilute nitric acid, acts with vehemence on the metal, reducing it speedily and completely to oxide, which on evaporation of acid, is left a greenish-white residuum

2. Dilute sulphuric acid, blackens it almost immediately, oxidizes very slowly.

3. Dilute hydrochloric (or muriatic) acid, blackens and oxidizes the surface more speedily than sulphuric acid. In both these acids, the oxide fall away in black powder.

4. Caustic potassa, in a deliquescent state, blackens it immediately, acts with energy and dissolves it into an inky mass. Tin is acted on in the same manner and with equal facility; bismuth similarly affected but more slowly; cadmium merely tarnished. No perceptible effect on silver, lead, antimony, iron, copper; neither sal. soda, nor carb. soda in solution has any action on the metal.

5. Solution of nitre, with alum and common salt, tarnishes it slowly. After twelve or fifteen hours the surface is blackened and eroded. As neither solutions of alum or salt appear to effect it, this action is doubtless due to the nitre.

6. Tartaric acid, in solution has no effect upon it, nor upon tin, bismuth, and copper, but it tarnishes antimony, iron, cadmium and lead.

7. Lemon juice (citric acid) tarnishes it very slowly; at first imperceptible, but after two days the surface is darkened. It has precisely the same effect on tin.

8. Acetic acid, diluted.—After two hours the metal is slightly tarnished; after two days considerably darkened; effect nearly same as lemon juice. Same action upon tin.

9. The same, heated, but not to boiling. Action immediate, rendering the metal black as coal in five minutes. Same effect on tin.

(In all the previous cases the fluids were cold.)

10. Oxalic acid, (in solution) tarnishes it considerably in two hours. After twelve hours the surface is of a deep black. Tin *appears* to be less affected. (The oxidation was not facilitated by heating this acid, but the heat was not prolonged.)

Where tin and cheoplastic metal were compared, they were placed in separate vessels, remaining of course the same length of time. But in most of the tests the time was not particularly noted, though in no case continued over about two days. It would be interesting to apply similar tests for several days or weeks, and then examine by aid of a strong magnifier, the depth of oxidation upon the surfaces. In the above I feel satisfied there could have been little difference in amount of oxidation on the two metals, but I think prolonged tests would prove, what theory would indicate, a greater facility and extent of action upon the *alloy* than upon pure tin.

It is needless to remark, that none of these re-agents have any action on platina or gold. All are liable to find their way into the mouth, either introduced with ingesta, or generated by decomposition of particles of food and other matters, or present in the secretions. Chemical analysis has already revealed in the saliva alone, when in acid conditions, the presence of acetic, lactic, hydrochloric or muriatic, oxalic and uric acids; the most active of which (so far as concerns cheoplastic metal) viz: hydrochloric acid is generated in cases of ordinary indigestion and simple gastric derangement from impropriety or excess in diet, to say nothing of its being thrown up in large quantity in eructations from the stomach. Of the saliva too, recollect its strong affinity for oxygen which it so readily absorbs from the air to impart again to other substances, especially metals and by which they are so liable to oxidation in the mouth.

The disposition of oxygen to unite with decayed particles of food, gives rise to the formation of acids according to the nature of the substances, whether nitrogenous, or non-nitrogenous. Dr. Samuel L. Mitchell, as long ago as 1796, drew attention to this subject in his letters to Dr. Hope of Edinburgh, wherein he illustrates the formation of septic or nitric acid in the mouth by the combination of oxygen with the septon, as he calls it, resulting from the remains of food—whether animal or vegetable containing nitrogen. "Nitric acid," says Prof. Graham in his *Chemistry*, "is largely produced by the oxidation of organic matters during putrefaction in air, when an alkali or lime is present," which is precisely the case in the mouth, the saliva in its ordinary state containing, in excess, alkali and lime. Prof. Harris, in his *Principles and Practice of Dental Surgery*, fifth edition, informs us that even gold plate, if alloyed with platina, (which alloy he says "is readily acted on by nitric acid") is liable to corrosion by the septic or nitrous acid which is formed in the mouth.

It is unnecessary to enumerate the various acids, alkalies, salts, or gases, which, whether from ingesta or secretions, find their common laboratory in the mouth, nor the reagents which their multiform combinations and reactions are liable to produce, capable of corroding the base metals, especially a compound like this cheoplastic alloy. We have already seen quite enough to invalidate the testimony of any parade of witnesses, however numerous or imposing, who assume to assert that it is "*as pure to wear as the gold commonly used*," that its "*non-corrosiveness is equal to gold or platina*," and that "*no acids that can ever come in contact with it in the mouth, and none of the secretions of the mouth can have any effect upon it.*"

It is, to say the very best for it, nothing more than tin rendered stiff, harsh and brittle, by the admixture of other metals, without modifying for the better, or disguising any of its chemical properties, or adding thereto a single virtue, and which, instead of "resisting oxidation" as claimed, is acted upon by all of the mineral and most of the vegetable acids, as well as by alkali and certain salts.

But this is not the worst, for I think it will appear that this vaunted *chemical union* of the other metals with the tin renders the alloy chemically inferior to pure tin, and consequently more objectionable for dental purposes. Of the adulterations—antimony, bismuth, and silver—the latter is certainly of no *advantage*, unless to *talk about.* And chemically considered, it is obnoxious, owing to galvanic action and greater liability to oxidation, induced by contact or combination of metals so different in electrical properties as silver and tin. It is well known that, if two such metals, the one negative, (like silver) and the other positive, (as tin) in respect to each other, are submitted in contact to an acid having an affinity for both or either, galvanic action is brought in play, which causes the acid to act with greater energy on the positive or more oxidable metal than it otherwise would. So of an alloy,—which is but a *contact of atoms*, presenting a mixture of positive and negative elements—the difference in electrical conditions of its components, by inducing galvanic action, increases the affinity of the acid for the positive atoms or particles.

With reference to this augmented susceptibility of alloys to oxidation in consequence of galvanic agency, there is reason for belief that it is greatest where *several* metals enter into the compound, although the absolute proportion of the metal alloyed, remains the same. Thus Dr. James Robinson (in a paper on the alloys of gold, 1852,) records the experiment of submitting three different specimens of fourteen carat gold, one alloyed with copper, one with silver, and one with copper and silver, each weighing $24\frac{1}{2}$ grains, to the action of nitric acid, in which the first lost $4\frac{1}{2}$ grains, the second $1\frac{1}{15}$ grain, and the third nearly $8\frac{1}{2}$ grains; whence he concludes that "gold alloyed with one metal is less liable to corrosive action, than an alloy which consists of the *three* metals."

This objection to the silver used in Dr. Blandy's metal is alike applicable to the bismuth and antimony; although as to bismuth I do not think that it would be of much moment if unassociated with the silver and antimony, since the electrical differences between bismuth and tin are so slight as to induce comparatively little electrical excitation.

But to antimony there is a graver objection. It is a poisonous metal, nearly allied to arsenic, with which, in chemistry, it forms a distinct class—the oxides of each corresponding to those of the other, and both remarkable for forming strong acids with oxygen. The oxide of antimony operates on the system as an irritant poison—it is the active base of tartar emetic. The action of nitric acid converts it into antimonic acid, a strong acid forming various salts with metallic oxides. The salts of antimony afford some of the most active and dangerous preparations in medicine; the powerful effect of tartar emetic, in small doses, taken internal, and also on skin and cellular tissue, as a particular ointment, is well known. Besides, antimony is rarely free from arsenic, with which it is naturally associated, and so great is their affinity that the latter finds its way into most of the pharmaceutical preparations of the former. "According to Serullas," says Prof. Bache, "all the antimonial preparations except tartar emetic, and butter or serquichtoride of antimony, contain a minute portion of arsenic." If this is the case after passing the ordeal of the dispensary, and the transformation into various salts, etc., what are we to expect of the crude metal?

But what effect this antimony, as contained in Dr. Blandy's compound, would have upon the mucous surfaces of the mouth, and with susceptible constitutions, must be left for time to determine. We can only for the present admonish the profession, in the Doctor's own language, of the "great danger of using impure metals in the mouth, * * * *as the injury will only be discovered when irreparable.*"

As a base for artificial teeth, pure tin has every advantage which can be justly claimed for this alloy (except perhaps that of stiffness) and being innocuous in the mouth, however objectionable in other respects, should be preferred to it in this style of work by those who consult consequences. Moreover, if desired, any degree of stiffness and elasticity could be bestowed by a small proportion of bismuth only, of which much less effects the same end than of antimony, without the danger. The surety and facility of obtaining it perfectly pure for the purpose, is also an important consideration.*

From experiments made some months ago, I am convinced that this single metal with tin gives a better flow, a more uniform surface, and is less liable to flaws and shrinkage, without association with antimony or other admixture, than with it. It whitens tin and gives it a very clean, handsome appearance. It may be used in the proportion of one part bismuth, to from ten to twenty parts tin: for ordinary use say one to twelve, which (to borrow the technicality of gold) would make the tin over twenty-two "carats" fine. For solder a larger proportion of bismuth will be required. As block tin is liable to contamination, as by lead, antimony etc., the best *grain tin* should be obtained. Or the metals properly refined, might be supplied by our dental furnishing houses.

But let us here, once for all, urge the fact that for all permanent work, gold and platina are the only reliable and unexceptionable metals.

If, however, *tin* is to be used let it be called "tin;" for although reduced to a certain standard by bismuth, or any other metal, or metals, it is still as much entitled to the appellation as the ordinary gold plate is to that of "gold." Let it then, wherever used, be known by its true name, in and out of the profession, as becomes honorable professional men, due in candor and manly dealing towards their patients. For it is better for the dentist to come out frankly, at once, than after evasion and quibbling have to admit the fact to an intelligent patient, or than to deceive a stupid one; better for the profession voluntarily to proclaim the truth to the public than to have the public discover it for themselves, and therein convict the profession of duplicity.

---

* "It may be purified from all contaminating metals, by dissolving the bismuth of commerce in diluted nitric acid, precipitating the clear solution by adding water to it, and reducing the white powder thus obtained (sub-nitrate of bismuth) with black flux."—*United States Dispensatory.*

Discarding, therefore, all names of equivocal import, and all secret and patent admixtures, whether alloys of tin or other base metals, which, like the one under consideration, present themselves *as substitutes for gold or platina*, let this new fangled "*Cheoplasty*" take its place among the "Royal Succedaneums" of the past—to mark the era of the most extraordinary imposition that has been attempted in the profession since the days of the Crawcours.

Nashville, Ten., May, 1858.

---

### ABSCESS OF MAXILLARY SINUS.

Having been solicited by quite a number of my professional friends, to give a short history of a rare case of Abscess of the *Maxillary Sinus*, which came under my treatment about one year since, I will at once proceed to detail, without further preliminary:

The patient was a lady, about twenty-four years of age, of rather active temperament, good general health, yet not of very great physical strength. About five months before I saw her, the left side of her face commenced troubling her, and at times the pain was very severe, continually growing more and more painful for about four or five weeks. During this time all of the left side of the face became very much swollen. The left eye suffered very much—the globe of the eye becoming very much inflamed—the muscles so swollen as to close over it. Vision was gone.

The nares also was closed by the swelling of the tissues, a raging fever spread over the whole system, entire prostration and confinement to her bed, was of course, the result. She lay in this condition most of the time for four months, having some treatment by the family Physician. During this time a very small accumulation of pus and other matter appeared directly below the eye. This she opened with the point of a pin, after which the severe pain left her for a short time. But as matter accumulated there would be some discharge, of pus but little, but more of a water like appearance. This opening would *appear* to close up entirely, sometimes, and then break out again. This was the condition I found the patient in.

By examination I found the abscess to be formed in the *maxillary sinus*, in the upper part, and immediately below the eye. I introduced an instrument directly under the eye passing through the floor of the orbit, directly into the antrum, without any obstruction of an osseous nature, found several pieces of necrosed bone which had formed the floor of the orbit, but very little discharge from this opening, no pus at all. Next removed the second bicuspid and first molar teeth, of the same side. Found no unusual appearance at the extremity of their fangs. Found that the fangs *did not* penetrate the antrum, that there was about one-fifth of an inch of firm healthy bone between the extreme point of the roots of those teeth and the *maxillary sinus*. From the alveolar cavity of the bicuspid I made a free opening into the antrum, of about one-third of an inch diameter. Through this there was a very large quantity of thick pus, which was not limpid enough to pass through a smaller opening which I made first, also some small pieces of bone. From the quantity of pus discharged I think that it completely filled the cavity.

At this stage, the patient being so much exhausted, I syringed out freely with tepid water and a little castile soap, and let her rest for two days.

Second sitting—found the face much less swollen, and the patient comparatively convalescent. Removed about one half of the left nasal bone, which was necrosed and through this opening into the nares some half dozen small pieces of bone, from one-eighth to one-half inch in length. After which I syringed all the parts freely with tepid soft water. Then prepared a pleget of linen wound firmly around a bit of Hickory wood, this I pressed firmly into the opening through the maxillary bone, as I wished this to be kept open until the other parts were healed.

But I fear that I am making this altogether too lengthy and will stop short merely saying that the parts were bathed freely every day as before mentioned, with some other mild treatment, and the patient recovered in about six weeks. Recovering her sight entirely. She is now well.

P. S. Let me say that the other side of her head and face had commenced swelling and paining her in the same way that this began. Most respectfully submitted,

OBERLIN, May 24, 1858. W. B. INGESOLL.

---

### CIRCULAR.

The "American Dental Convention" will hold its Fourth Annual Session at Cincinnati, Ohio, on Tuesday the 3d day of August next, and as this Convention is open to "*all* practicing Dentists," it is expected you will be present. The consequence will be the elevation of the whole Dental Profession, proving of incalculable benefit to your patients, they knowing which will also expect you to be present. We therefore trust you will not absent yourself therefrom, but come, and as the subjects for discussion are inclosed, come prepared; and by writing out, concisely, your experience on the various topics, it will greatly facilitate the business of the Convention, and add greatly to the amount of knowledge derived by the meeting.

The following resolution was adopted:

*Resolved*, "That the Corresponding Secretary request all persons having anything new and useful to the Dental Profession, to present the same at the next meeting of the Society."

Believing you will make every exertion to come,

I am truly yours,

ISAIAH FORBES, *Cor. Sec'y.*

1.—The best means of securing and preserving good teeth.
2.—Treatment of exposed nerves.
3.—Mechanical Dentistry.
4.—Filling teeth.
5.—Miscellaneous.

We fully agree with the opinion expressed by the "Dental Review," that "To add words to the above excellent circular would be entirely superfluous. An observance of the suggestions contained in it will make the meeting an eminently successful one."

Those who have been in the habit of meeting from time to time with their brethren will not permit any ordinary obstacle to prevent their being present, for they will know the good times they have had in days by-gone, and the great value those meetings have been to them. To those who do not attend we would just say, you have no conception of your loss, both socially and professionally. If every member of the profession felt as he should do, there would be an outpouring of from one to two thousand strong.

Gentlemen we want, very much, to greet a thousand or more here, on the first Tuesday of August next.—Ed.

St. Louis, April 13, 1858.

Dear Sir:—The annual Meeting of the "Western Dental Society," will meet in Quincy, Illinois, on the 3d Tuesday, the 20th of July next.

The object of the meeting of the Society will be the elevation of the profession in the West, and the improvement of its members. It is expected that *you* will be present to aid in the good cause.

The following subjects have been presented by the committee, for discussion.

1.—Difficult Dentition.

2.—Cause and treatment of the irregularity of the teeth.

3.—Best method of treating dentine when the pulp is exposed, or destroyed by natural causes.

4.—Filling Teeth.

5.—Best manner of inserting partial sets with clasps.

6.—Relative merits and demerits of the various modes of constructing artificial dentures.

The Committee also urge upon members and visitors having reports of cases, casts, cuts, diagrams, or specimens of operations or work, with new instruments, tools, or fixtures intended to promote the interest or facilitate the practice of our profession, to present them for inspection before the Society.

Trusting that you will be with us,

I remain yours,

H. J. McKellops, *Cor. Sec'y.*

We expect to see the profession out in its *might* at Quincy, on the 20th of July.

The people in the West generally do not do things by fractions, they take whole numbers and large ones at that. The Dental profession is no exception to this rule; they will be there, every man with a *stone* in his hand.

There is a rich programme of subjects for discussion, and we doubt not full justice will be done to them.

---

### WOODEN TEETH.

I once saw a pivot tooth, made of oak, then dipped in melted sperm and thus enameled, worn by a Rev. D. D., it looked quite well. I had a call recently from one of the sons of Erin, who desired me to reset a tooth, made of hickory, and held in situ by silk ligatures, made and adjusted as he told me, "by a nice dentist in Cincinnati, shure.

H. E. Peebles.

Will the "Register," give us a name "for the above new process," if not "a report." "Cheoplasty, Teoplasty," umph! —*Am. Dental Review.*

Dr. A. M. Leslie:—*Dear Sir:* In looking over the minutes of the proceedings of the Western Dental Society, I see that we "adjourned to meet at Quincy, Ills., on the 3d Tuesday (22d) in July, 1858," but it seems that the 3d Tuesday falls on the 20th and not the 22d, as already announced in the Journals.

Please make the correction in the May No. of the Review and call attention to it. Respectfully,

H. E. Peebles, *Ch'n Com.*

St. Louis, April, 1858.

Will the Register, News-Letter, and Journal please notice the above correction in their next issues.—*Ibid.*

### LOCAL ANÆSTHESIA.

BY MONS. PIEDOGUEL.

Cauterization by caustics is an operation frequently employed in surgery, but as it is very painful, patients are repugnant to submit to it. I have made some experiments to try to produce cauterizations without causing much pain, and I have fixed upon the following means :

When we mix Vienna Powder (de la powdre de Vienne) and hydrochlorate of morphine, by means of a liquid, we obtain a paste which, applied upon the skin, produces an eschar, without causing pain.

The mixture of three parts of Vienna Powder (quick lime and caustic potash) and of one part of hydrochlorate of morphine should be made intimately and without wetting, then add chloroform, alcohol or water, so as to obtain a thick paste, which is applied to the skin by means of cerecloth of diachylum ; after five minutes of this application, the skin which the cautery covers becomes of a dead white color ; five minutes later it forms at its circumference a little whitish edemacious pod, and at the end of fifteen minutes the skin is brown, parched, charred, and then the thickness of the eschar increases with the duration of the application and becomes nearly equal to the paste employed. As to the diameter, it is always greater than that of the caustic, but that depends on the mode of application.

By adding a little gum to the paste, it may be made up in small disks one centimeter in diameter by four or five millimetres in thickness ; by heat they become very hard, but they act less promptly, and it is necessary to moisten them with water before they are applied.

The hydrochlorate of morphine may be employed, mixed in the same proportions, (a quarter,) with powder of cantharides ; then vesication is produced without causing pain. A granime of hydrochlorate of morphine thus mixed, has after an applicrtion of ten hours, occasioned only a slight and transient somnolence; but it is useless to employ so strong doses —from thirty to forty centigrammes suffice for a cautery or a vesicatory.

The hydrochlorate of morphine confines its action to the part upon which the caustic is applied. There is no absorption—no intoxication; it is then a local *anesthesia*, since we can attain the same results by using water or alcohol.

As a physician, I have been able to employ this means only for establishing issues, and I have applied it to all parts of the body. In surgery it can be made useful in many circumstances, to obviate pain and destroy diseased parts. Professor Jobert de Lamballe has had the kindness to permit me to make use of it in his practice, and we have applied it to scrofulous congestions of the neck, to an encephaloid cancer of the foot, and I have already employed it for the purpose of destroying sympathetic vegetation; but at this time I limit myself to a statement of what I have obtained for cauterics and vesicatories.—*L. Art Dentare for April*, 1858, *translated by Coats-Kinney.*

